# A Higher Prevalence Rate of *Campylobacter* in Retail Beef Livers Compared to Other Beef and Pork Meat Cuts

**DOI:** 10.3390/ijerph10052058

**Published:** 2013-05-21

**Authors:** Aneesa Noormohamed, Mohamed K. Fakhr

**Affiliations:** Department of Biological Science, The University of Tulsa, Tulsa, OK 74104, USA; E-Mail: aneesa-noormohamed@utulsa.edu

**Keywords:** *Campylobacter*, antibiotic resistance, prevalence, retail beef, beef livers, retail pork, foodborne pathogens

## Abstract

The objectives of this study were to determine the prevalence of *Campylobacter jejuni* and *Campylobacter coli* in retail beef, beef livers, and pork meats purchased from the Tulsa (OK, USA) area and to further characterize the isolates obtained through antimicrobial susceptibility testing. A total of 97 chilled retail beef (50 beef livers and 47 other cuts), and 100 pork samples were collected. The prevalence of *Campylobacter* in beef livers was 39/50 (78%), while no *Campylobacter* was isolated from the other beef cuts. The prevalence in pork samples was 2/100 (2%). A total of 108 *Campylobacter* isolates (102 beef livers isolates and six pork isolates) were subjected to antimicrobial resistance profiling against sixteen different antimicrobials that belong to eight different antibiotic classes. Of the six pork *Campylobacter coli* isolates, four showed resistance to all antimicrobials tested. Among the beef liver isolates, the highest antibiotic resistances were to tetracyclines and β-lactams, while the lowest resistances were to macrolides, aminoglycosides, lincosamides, and phenicols. Resistances to the fluoroquinolone, macrolide, aminoglycoside, tetracycline, β-lactam, lincosamide, and phenicol antibiotic classes were significantly higher in *Campylobacter coli* than *Campylobacter jejuni* isolates. Multidrug Resistance (MDR) among the 102 *Campylobacter* (33 *Campylobacter jejuni* and 69 *Campylobacter coli*) beef liver isolates was significantly higher in *Campylobacter coli* (62%) than *Campylobacter jejuni* (39%). The high prevalence of *Campylobacter* in retail beef livers and their antimicrobial resistance raise concern about the safety of these retail products.

## 1. Introduction

*Campylobacter* species are Gram-negative bacteria that are considered the second leading cause of foodborne disease in the US [1]. *Campylobacter* causes gastrointestinal diseases characterized by diarrhea (which is often bloody), abdominal cramping, fever, and vomiting [2,3]. Campylobacteriosis is usually self-limiting, but in rare cases it has been shown to trigger Guillain-Barré syndrome, an autoimmune disease of the peripheral nervous system that can lead to paralysis [4,5]. The most common sources of *Campylobacter* are chicken and turkey products [6]. Most infections occur from the improper handling or consumption of raw or undercooked meat. While *Campylobacter jejuni* is more prevalent in human infections than *Campylobacter coli,* 95% of infections are usually due to one of these two species [7,8]. 

The macrolide erythromycin is known to be used in the treatment of human campylobacteriosis. Fluoroquinolones like ciprofloxacin are used to treat enteritis, while aminoglycosides are commonly prescribed for the treatment of systemic infections [4,9]. Reports of emerging multidrug resistant *Campylobacter* isolates in recent years may complicate the treatment of human infections [10,11].

Antimicrobial resistance in foodborne pathogens is of significant concern to human health [11,12]. This is due to the fact that many of the drugs that are used to treat human infections are used in animal husbandry as prophylactics and feed supplements, which have been shown to the selection of resistant isolates that may affect human health if they get into the food chain [13]. Infections with drug-resistant *Campylobacter* have been associated with increased mortality and morbidity [13]. There has been a correlation shown between the increased use of certain antibiotics and increased resistance to these antibiotics [13,14].

Most of the available studies are concerned mainly with the prevalence of *Campylobacter* in retail beef and pork while a limited number of them discussed the antimicrobial resistance of the isolates to few antimicrobials. While *Campylobacter* prevalence is relatively high in cattle and pigs at the farm level, it appears that, on the other hand, most of *Campylobacter* prevalence studies on retail beef and pork meat is showing a lower incidence [5,11,15,16,17]. The number of studies discussing *Campylobacter* in beef livers is very limited in the literature [18]. The objectives of this study were to determine the prevalence of *Campylobacter jejuni* and *Campylobacter coli* in retail beef, beef livers and pork meats obtained from the Tulsa, OK area grocery stores and to further characterize the isolates obtained through antimicrobial susceptibility testing.

## 2. Experimental Section

### 2.1. Samples Collection and Campylobacter Isolation

A total of 97 retail beef (50 beef livers and 47 other cuts) and 100 pork samples were purchased from several Tulsa area grocery stores on weekly bases from January to June 2010. Beef samples other than livers were from different cuts such as, steak, stew, shoulder, and bone in. Samples were selected to be variable as possible with different expiration dates and production codes. Meat samples were purchased from nine grocery stores that belong to six different chains at variable locations in the city. The collected beef samples belonged to nine different brands while the pork ones were from seven brands. The retail meat samples were transported to the laboratory in ice boxes and processed immediately upon arrival. Samples were rinsed with Buffered Peptone Water (BPW; EMD, Gibbstown, NJ, USA) in sterile plastic bags (VWR Scientific, Radnor, PA, USA) and massaged briefly by hand for five minutes. Next, 10 mL of the rinsate was added to 10 mL of 2× Bolton broth supplemented with blood and the appropriate antibiotic supplementation. The enrichment was incubated at 42 °C for 48 h and then plated onto *Campylobacter* Charcoal Desoxycholate Agar (CCDA; Remel, Lenexa, KS, USA) plates with the appropriate antibiotic supplementation. The plates were incubated at 42 °C for 48 h in gas jars containing microaerophilic gas-generating kits (Mitsubishi Gas Chemical, New York, NY, USA). Four to six suspect *Campylobacter* colonies of each sample from the CCDA plates were then transferred onto Mueller-Hinton (MH; Difco, Sparks, MD, USA) agar plates supplemented with blood and incubated at 42 °C for 48 h under microaerophilic conditions, then purified by sub culturing and kept at −80 °C freezer for preservation until subjected to molecular identification.

### 2.2. DNA Extraction

Bacterial DNA extracts used in polymerase chain reaction (PCR) were prepared from *Campylobacter* cultures using the single-cell lysing buffer (SCLB) method [19]. Isolates were removed from −80 °C storage, struck to Mueller-Hinton agar (MH; Difco), and incubated at 42 °C for 48 h under microaerophilic conditions. One colony was picked from the plate and suspended in 40 µL of SCLB solution in a 0.2 mL microtube. The SCLB solution consisted of 10 µL of 5 mg/mL proteinase K (Amresco, Solon, OH, USA) and 1.0 mL of TE buffer (10 mM Tris-HCl (J.T. Baker, Phillipsburg, NJ, USA) and 1 mM EDTA (Fisher, Fair Lawn, NJ, USA)). The cells were lysed by heating at 80 °C for 10 min, followed by cooling to 55 °C for 10 min, using a Mastercycler Gradient thermocycler (Eppendorf, Eppendorf, Germany). The suspension was diluted 1:2 in double distilled water and centrifuged in a Microfuge (Clover Laboratories, Waterville, OH, USA) at 4,500 × g for 30 s to remove cellular debris. The supernatant was used as DNA template for PCR. All DNA extract samples were stored at −20 °C.

### 2.3. PCR Identification

All *Campylobacter* suspect isolates were tested for the identification of *Campylobacter* genes by multiplex PCR reaction using primers specific for *Campylobacter jejuni* and *Campylobacter coli* [20] (Table 1). The PCR was carried out in 25 µL reactions. Each 25 µL reaction contained 12.5 µL *Taq* Polymerase Master Mix (Qiagen Inc., Valencia, CA, USA), 7.5 µL sterile water (Qiagen), 1 µL (25 pmol) each primer (IDT, Coralville, IA, USA), and 3 µL of template DNA. The cycling conditions were set as follows on the Mastercycler Gradient thermocycler, initial denaturing at 95 °C for 5 min followed by 30 cycles of denaturing at 94 °C for 1 min, annealing at 52 °C for 1 min, and extension at 72 °C for 2 min, followed by final extension at 72 °C for 10 min (modified from Konkel, *et al.* [21]). At the completion of the Polymerase Chain Reaction, reactions were held at 4 °C until gel electrophoresis. The expected amplicon sizes were 160 bp for *C-1* gene, 400 bp for the *cadF* gene, and 894 bp for *ceuE* gene (Table 1). *Campylobacter jejuni* ATCC #33560, and *Campylobacter coli* strain #96121033 (Oklahoma Animal Disease Diagnostic Laboratory, OSU, Stillwater, OK, USA) were used as positive controls, and sterile water was used as a negative control.

**Table 1:** A list of PCR primers used for *Campylobacter jejuni* and *Campylobacter coli* identification and their corresponding amplicon sizes and references.

Gene	Size (bp)	Primer sequences	Species	References
*cadF*	400	F 5′-TTGAAGGTAATTTAGATATG-3′	*Campylobacter coli & Campylobacter jejuni*	[20,21]
		R 5′-CTAATACCTAAAGTTGAAC-3′		
*ceuE*	894	F 5′-ATGAAAAAATATTTAGTTTTTGCA-3′	*Campylobacter coli*	[20,22]
		R 5′-ATTTTATTATTTGTAGCAGCG-3′		
*C-1*	160	F 5′-CAAATAAGTTAGAGGTAGAATGT-3′	*Campylobacter jejuni*	[20,23]
		R 5′-GGATAAGCACTAGCTAGCTAGCTGAT-3′		

### 2.4. Antimicrobial Susceptibility Testing

Antimicrobial susceptibility testing was performed using the agar dilution plate technique for a total of 108 *Campylobacter* isolates against sixteen antimicrobials belonging to eight different antibiotic classes (Table 2). Isolates were grown on Mueller-Hinton (MH) agar (Difco) supplemented with 5% laked horse blood (Hemostat Laboratoties, Dixon, CA, USA) and incubated for 48 h at 42 °C at microaerophilic conditions. Cultures were then added to Mueller-Hinton broth (Difco), adjusted to turbidity equal to a 0.5 McFarland standard, and inoculated onto 6-inch MH agar plates supplemented with 5% blood and antimicrobials at different concentrations (Table 2) including the breakpoint established for each antimicrobial according to the Clinical and Laboratory Standards Institute (CLSI) (ciprofloxacin, erythromycin, doxycycline, and tetracycline), National Antimicrobial Resistance Monitoring System (NARMS) (gentamicin, clindamycin, azithromycin, and nalidixic acid), and other published articles (ampicillin, streptomycin, and chloramphenicol [24], amoxicillin [25], cephalothin [26], kanamycin [27], tilmicosin [28], and oxytetracycline [29]) . *Campylobacter jejuni* ATCC #33560 was used as a quality control strain. The plates were incubated at 42 °C for 48 h under microaerophilic conditions. The plates were read for growth or no growth and denoted as resistant or susceptible, respectively according to the breakpoints for each of the sixteen tested antimicrobials (Table 2). Multidrug Resistance (MDR) was defined as resistance to three or more antibiotic classes [30]. Kruskal-Wallis test was used to compare resistance of *Campylobacter jejuni* and *Campylobacter coli* to the 16 tested antimicrobials. Fisher’s exact test was used to compare MDR between the two species.

**Table 2:** A list of the sixteen tested antimicrobials, their classes, the concentrations used for susceptibility testing, and the breakpoints used for each antimicrobial.

Antimicrobial Class	Antimicrobial	MIC Range (µg/mL)	Breakpoint (µg/mL)
β-lactams	Amoxicillin	16–256	32
	Ampicillin	16–256	32
	Cephalothin	16–256	32
Aminoglycosides	Gentamicin	4–64	8
	Kanamycin	32–512	64
	Streptomycin	48–512	64
Quinolones	Nalidixic Acid	32–512	64
Fluoroquinolones	Ciprofloxacin	2–32	4
Macrolides	Azithromycin	4–64	8
	Erythromycin	16–256	32
	Tilmicosin	4–64	8
Lincosamides	Clindamycin	4–64	8
Tetracyclines	Doxycycline	4–64	8
	Oxytetracycline	1–16	2
	Tetracycline	8–128	16
Phenicols	Chloramphenicol	16–256	32

## 3. Results and Discussion

### 3.1. Prevalence of Campylobacter in Beef and Pork

The overall prevalence of *Campylobacter* (*Campylobacter jejuni* and *Campylobacter coli*) in beef livers was 39/50 (78%), whereby 13/50 (26%) of the samples was contaminated with *Campylobacter jejuni*, 24/50 (48%) of the samples was contaminated with *Campylobacter coli*, and 2/50 (4%) of the samples was contaminated with both *Campylobacter jejuni* and *Campylobacter coli*. None of the 47 other beef cuts contained *Campylobacter*. The prevalence of *Campylobacter* (*Campylobacter jejuni* and *Campylobacter coli*) in pork was 2/100 (2%). The pork samples contained only *Campylobacter coli* (2/100) and no *Campylobacter jejuni* was found. 

The low prevalence of *Campylobacter* in retail pork meat (2%) is not surprising and in relative agreement with previous studies. Zhao *et al.* [6] found that the rate of *Campylobacter* contamination in pork chops was 0.5%. Hong *et al.* [16] reported a *Campylobacter* prevalence of only 1.6% in pork meat where Wong *et al.* [15] found a relatively higher prevalence of 9.1%. Both of the pork samples contained *Campylobacter coli*. Most of *Campylobacter* contaminations of pork have generally been found to be *Campylobacter coli* [31,32,33]. 

The overall prevalence of *Campylobacter* in beef livers in our study was 39/50 (78%) while no *Campylobacter* was isolated from the other beef cuts. Despite their limited number, previous studies have found beef livers to be contaminated with *Campylobacter*. Strachan *et al.* [18] found that 69% (22/32) of their beef liver samples were contaminated with *Campylobacter*. Kramer *et al.* [32] found that 54.2% of ox livers they tested were contaminated with the bacteria. On the other hand, Enokimoto *et al.* [34] found a *Campylobacter* prevalence of only 5% in the beef livers they tested but their beef liver samples were picked up at a meat slaughter center in Japan right after the cattle slaughtering and was surface sterilized to exclude any bacteria on the surface. The high prevalence of *Campylobacter* in retail beef livers is alarming and might be due to cross contamination since the livers are recovered from several cows and possibly piled up together. Ghafir *et al.* [17] suggested that the high level of recovery of *Campylobacter* from livers is probably due to the fact that the liver surface stays moist, which might protect this foodborne pathogen. Fecal carriage of *Campylobacter* by the slaughtered cows is a possible source of contaminating beef livers in slaughter houses. Liver location makes them easily prone to bile contamination. The risk of this high prevalence of *Campylobacter* in beef liver could be magnified by cooking livers lightly to avoid overcooking undesired taste. 

Out of the 39 beef liver samples that were positive in our study 13 (33%) were *Campylobacter jejuni* and 24 (62%) were *Campylobacter coli* and two samples were contaminated with both species. Kramer *et al.* [32] found in their study that 49% of their *Campylobacter* isolates from beef livers were *Campylobacter jejuni* and 2.1% were *Campylobacter coli*. Ghafir, *et al.* [17] found that in their beef samples, all of the isolates were *Campylobacter jejuni*. In a fecal samples study, Nielsen *et al.* [31] found 90.9% of the isolates from cattle were *Campylobacter jejuni* and 6.8% were *Campylobacter coli*. In contrast to those reports, our study showed higher numbers of *Campylobacter coli* than *Campylobacter jejuni*. Some of the differences in *Campylobacter* prevalence discussed above might be due to seasonal or geographic area variations. 

### 3.2. Antimicrobial Resistance Profiling

A total of 108 *Campylobacter* isolates (102 beef liver isolates and six pork isolates) were subjected to antimicrobial resistance profiling against sixteen different antimicrobials that belong to eight different antibiotic classes (Table 2, Table 3). Table 3 shows the percentage of resistance of the 102 *Campylobacter* isolates (33 *Campylobacter jejuni* and 69 *Campylobacter coli*) isolated from beef livers to the sixteen tested antimicrobials that belong to eight antibiotic classes. The percentage of resistance to the sixteen tested antimicrobials varied between *Campylobacter jejuni* and *Campylobacter coli* isolates. Among the beef liver isolates, resistance to fluoroquinolones (ciprofloxacin) was significantly higher in *Campylobacter coli* (62%) than in *Campylobacter jejuni* (39%) (Table 3). It is also shown in Table 3 that none of the tested *Campylobacter jejuni* isolates were resistant to all tested macrolides, while only 29% of *Campylobacter coli* were resistant. Twenty percent of the tested beef liver *Campylobacter coli* isolates was resistant to the three tested aminoglycosides while none of the *Campylobacter jejuni* isolates showed resistance (Table 3). Resistance to all tested tetracyclines was significantly higher in *Campylobacter coli* (97%) than in *Campylobacter jejuni* (73%). Resistance to phenicols, lincosamides, and β-lactams was significantly higher in *Campylobacter coli*, and there was no significant difference for resistance to quinolones between the two species (Table 3). Of the six *Campylobacter coli* pork isolates, four showed resistance to all antimicrobials tested and the other two were resistant to all of the antimicrobials tested except clindamycin, erythromycin, and gentamicin (data not shown). The distribution of the Multidrug Resistance (MDR) which was defined as resistance to three or more antibiotic classes among the 102 *Campylobacter* (33 *Campylobacter jejuni* and 69 *Campylobacter coli*) beef livers isolates was significantly higher in *Campylobacter coli* (62%) than *Campylobacter jejuni* (39%) (Table 3). 

**Table 3:** Antimicrobial resistance of the 102 *Campylobacter* beef livers isolates against 8 different antibiotic classes.

Antimicrobial Resistance					
Antibotic Classes	Antimicrobials	Beef livers			
		*C. jejuni* * np/n (%)	*C. coli* * np/n (%)	Significance	Total * np/n (%)
**β-lactams**	Amoxicillin	33/33 (100)	69/69 (100)	*P*-Value < 0.100	102/102 (100)
	Ampicillin	13/33 (39)	61/69 (88)	*P*-Value < 0.001	74/102 (73)
	Cephalothin	31/33 (94)	69/69 (100)	*P*-Value < 0.040	100/102 (98)
	All three tested	13/33 (39)	61/69 (88)	*P*-Value < 0.001	74/102 (72)
**Aminoglycosides**	Gentamicin	0/33 (0)	16/69 (23)	*P*-Value < 0.003	16/102 (16)
	Kanamycin	6/33 (18)	36/69 (52)	*P*-Value < 0.001	42/102 (41)
	Streptomycin	1/33 (3)	22/69 (32)	*P*-Value < 0.001	23/102 (23)
	All three tested	0/33 (0)	14/69 (20)	*P*-Value < 0.001	14/102 (14)
**Quinolones**	Nalidixic Acid	14/33 (42)	40/69 (58)	*P*-Value < 0.143	54/102 (53)
**Fluoroquinolones**	Ciprofloxacin	13/33 (39)	43/69 (62)	*P*-Value < 0.030	56/102 (55)
**Macrolides**	Azithromycin	1/33(3)	22/69 (32)	*P*-Value < 0.001	23/102 (23)
	Erythromycin	1/33 (3)	37/69 (54)	*P*-Value < 0.001	38/102 (37)
	Tilmicosin	2/33 (6)	24/69 (35)	*P*-Value < 0.002	26/102 (25)
	All three tested	0/33 (0)	20/69 (29)	*P*-Value < 0.001	20/102 (20)
**Lincosamides**	Clindamycin	1/33 (3)	16/69 (23)	*P*-Value < 0.011	17/102 (17)
**Tetracyclines**	Doxycycline	27/33 (82)	69/69 (100)	*P*-Value < 0.001	96/102 (94)
	Oxytetracycline	33/33 (100)	69/69 (100)	*P*-Value < 0.100	102/102 (100)
	Tetracycline	24/33 (73)	67/69 (97)	*P*-Value < 0.001	91/102 (89)
	All three tested	24/33 (73)	67/69 (97)	*P*-Value < 0.001	91/102(89)
**Phenicols**	Chloramphenicol	0/33 (0)	22/69 (32)	*P*-Value < 0.001	22/102 (22)
**MDR (resistant to 3 or more classes)**		13/33 (39)	43/69 (62)	*P*-Value < 0.001	56/102(55)

* np: No. of resistant isolates, n: no. of isolates tested.

Among the beef liver isolates, the highest antibiotic resistances were to tetracyclines and β-lactams, while the lowest resistances were to macrolides, aminoglycosides, lincosamides, and phenicols (Table 3). The highest antibiotic resistances of *Campylobacter* were to oxytetracycline, amoxicillin, cephalothin, doxycycline, tetracycline and ampicillin, while the lowest resistances were to gentamicin and clindamycin (Table 3). Ishihara *et al.* [35] found that 34% of their *Campylobacter jejuni* isolates and all three of their *Campylobacter coli* isolates were resistant to oxytetracycline. They found no ampicillin or erythromycin resistance among their isolates. Chartre *et al.* [11] found among their cattle isolates, 2.4% to gentamicin, 9% resistance to ampicillin, 18% to erythromycin, 44% to ciprofloxacin, 44% to nalidixic acid and 88.1% to tetracycline. Taremi *et al.* [36] found no resistance to erythromycin among their *Campylobacter* isolates. Ishihara *et al.* [35] found that 1.5% of their *Campylobacter coli* isolates were resistant to ampicillin, 32% were resistant to nalidixic acid, 48.5% to erythromycin, and 82% to oxytetracycline. In a study of pig farms, *Campylobacter coli* isolated from pigs were found to contain 20% resistance to ampicillin, 34% to nalidixic acid, 55% to erythromycin, and 79% to tetracycline [37]. Velazquez *et al.* [38] found that 99% of their isolates were resistant to cephalothin and 16.7% were resistant to ampicillin. 

As shown in Table 3 one can conclude that most of the isolates with the highest rates of antimicrobial resistance were *Campylobacter coli*. This has been seen previously, where *Campylobacter coli* carry on average more antimicrobial resistances than do *Campylobacter jejuni* [6,11,35,39]. Ge *et al.* [39] found no chloramphenicol resistance in *Campylobacter jejuni* and only 3% in *Campylobacter coli*. Our study also found no resistance in *Campylobacter jejuni* to chloramphenicol but *Campylobacter coli* had a 32% resistance rate. Their study also found that *Campylobacter jejuni* had 42% resistance rate to erythromycin while *Campylobacter coli* had 61% [39]. Our study showed similar pattern in regards to *Campylobacter coli* where it showed a 54% resistance to erythromycin but *Campylobacter jejuni* showed only a 3% resistance. Little *et al.* [40] showed that their *Campylobacter coli* isolates were resistant to erythromycin 16.7%). They found that their *Campylobacter coli* pork isolates had higher resistances to tetracycline, nalidixic acid, ciprofloxacin and erythromycin. Another study showed that the rates of resistance among *Campylobacter coli* to erythromycin was 25%, tetracycline was 96%, ciprofloxacin was about 97%, doxycycline was 98%, and nalidixic acid was 100% [16]. Among *Campylobacter jejuni* isolates they found resistances of erythromycin was 6%, tetracycline was 94%, ciprofloxacin and nalidixic acid was about 96%, and doxycycline was 98% [16]. 

In our study resistances to fluoroquinolones, macrolides, aminoglycosides, tetracyclines, β-lactams, lincosamides, and phenicols antibiotic classes were significantly higher in *Campylobacter coli* than *Campylobacter jejuni* isolates. While the overall prevalence of multidrug resistance in *Campylobacter* in our study is alarming, the fact that *Campylobacter jejuni*, the predominant species in human infections showed lower resistance to fluoroquinolones, and higher susceptibility to macrolides, and aminoglycosides is encouraging since human treatments are mostly dependent on these classes. 

## 4. Conclusions

The prevalence of *Campylobacter* in retail beef livers is significantly higher than in other beef and pork meat cuts. Multidrug resistance was generally higher in the *Campylobacter coli* isolates than in the *Campylobacter jejuni* ones. Beef livers should be cooked thoroughly before consumption and should not be fed raw to household pets. The high prevalence of *Campylobacter* in retail beef livers and their antimicrobial resistance seen in this study raise concerns about the use of antimicrobials and the safety of these retail products.
